# Research Progress in Hematological Malignancies: A Molecular Genetics Perspective

**DOI:** 10.3390/genes16060663

**Published:** 2025-05-29

**Authors:** Rina Kansal

**Affiliations:** 1 Molecular Oncology and Genetics, Diagnostic Laboratories, Versiti Blood Center of Wisconsin, Milwaukee, WI 53233, USA; rinakansal@msn.com; 2 Department of Pathology and Anatomical Sciences, The University at Buffalo, Buffalo, NY 14260, USA

This Special Issue in *Genes* presents articles on different types of hematological malignancies of interest to hematologists, oncologists, pathologists, and researchers involved in the care of patients with acute and chronic leukemias, lymphomas, and individuals with an underlying germline genetic predisposition to developing hematologic malignancies. These articles highlight the research progress in various aspects relevant to patients with hematological malignancies, including their diagnosis, prognosis, treatment, and prevention. Hematological malignancies are broadly classified according to the lineage of the neoplastic cells [1], with lymphoid neoplasms being the most common, followed by myeloid malignancies. 

## 1. Resistance to Bruton Tyrosine Kinase (BTK) Inhibitors During the Treatment of Chronic Lymphocytic Leukemia

Chronic lymphocytic leukemia (CLL) is a mature B-cell lymphoid neoplasm representing the most common leukemia in the Western world [2]. CLL accounts for one of three new cases of leukemia in the United States, where approximately 23,690 new cases of CLL and 4460 deaths from CLL are expected to occur in 2025 [3].

Over the last decade, Bruton tyrosine kinase (BTK) inhibitors have revolutionized the treatment of CLL and other mature B-cell lymphoid malignancies, including mantle cell lymphoma and Waldenström’s macroglobulinemia. This is because BTK’s role is integral in the antigen-activated B-cell receptor pathway, initiating the complex downstream signaling required for B-cell survival, proliferation, and differentiation. The *BTK* gene encodes a cytoplasmic non-receptor tyrosine kinase, a 659-amino-acid protein comprising five domains: the pleckstrin homology domain, the Tec homology domain, the Src homology domains, SH3 and SH2, and the kinase domain [4]. Mutations in *BTK* were initially identified as the cause of X-linked agammaglobulinemia, a rare primary immunodeficiency disorder characterized by deficient B-cell maturation (reviewed in [4]). The current standard-of-care treatment regimens for CLL include BTK inhibitors as a monotherapy or in combination [5]. Three BTK inhibitors currently approved for the treatment of CLL, ibrutinib, acalabrutinib, and zanubrutinib, covalently bind the cysteine 481 (C481) residue in BTK to irreversibly inhibit BTK signaling [6,7]. In contrast, non-covalent BTK inhibitors, such as pirtobrutinib and nemtabrutinib, reversibly bind to BTK at non-C481 sites and are designed to act on both unmutated and C481-mutant BTK [7,8]. However, the continued treatment of CLL with BTK inhibitors may lead to resistance through various mechanisms, including the development of resistance mutations at the binding sites in *BTK* and its downstream target gene, phospholipase C-γ2 (*PLCG2*) [9,10,11].

In the first article published in this Special Issue, Chirino et al. focused on describing the resistance mutations that occur after the treatment of CLL with BTK inhibitors, the alternative resistance mechanisms, and the impact on newer approaches to drug development [12]. The latter includes the development of protein degraders in drug therapies, which degrade the protein instead of inhibiting it [13,14]. In CLL, the BTK degrader NX-2127 degrades both unmutated and mutant forms of BTK [12,13,14,15].

## 2. Prognostic Risk Evaluation in Acute Myeloid Leukemia (AML) by a Comprehensive 12-Gene Metabolic Signature Model

The second article published in this Special Issue, by Zhai et al., pertains to evaluating prognostic risk in acute myeloid leukemia (AML) [16]. AML, a hematological malignancy classified as a myeloid neoplasm [1], is characterized by significant heterogeneity in its underlying genomic abnormalities, clinical tendencies for disease relapse, and unfavorable long-term outcomes [17]. The prognostic risk in AML is currently evaluated via the detection of cytogenetic and molecular genetic abnormalities, in conjunction with clinical information [18]. Several additional variables are continually being investigated to achieve a more precise assessment [19]. The metabolic aspects of AML are of particular interest, including those of leukemia stem cells (LSCs), which initiate AML at its onset and contribute to relapse [20]. The unique metabolism of LSCs, especially their reliance on mitochondrial oxidative phosphorylation, is closely linked to the genesis of AML [20,21]. In their research article, Zhai et al. described how they developed a comprehensive metabolic gene signature model that includes 12 metabolism-related genes to stratify prognostic risk and predict clinical outcomes in AML [16]. Their model was based on analyzing publicly available AML databases and requires validation in clinical studies [16].

## 3. Diagnosis of Molecular Genetic Abnormalities in Acute Leukemias by Reverse Transcriptase Quantitative Polymerase Chain Reaction (RTqPCR) Assays

The diagnosis and classification of hematological malignancies increasingly require genetic evaluation in clinical laboratories, particularly in acute leukemias [22]. In the research article by Pessoa et al., the authors identified the best endogenous genes to use as reference genes for the normalization step of a real-time reverse transcriptase quantitative polymerase chain reaction (RT-qPCR) assay [23]. This normalization step is crucial for obtaining reliable results in RT-qPCR assays, as reviewed in their study [23]. The authors prospectively selected peripheral blood and bone marrow samples from 49 adult and 25 pediatric patients with acute leukemia, including AML and acute lymphoblastic leukemia, for their study, as these samples are most often examined by molecular assays at diagnosis and during therapy for leukemia [23]. RT-qPCR assays were performed on RNA extracted from the clinical samples and 15 samples collected from healthy donors to determine gene expression levels, using the following six endogenous genes: *ABL1* (Abelson murine leukemia viral oncogene human homolog 1), *ACTB* (β-actin), *GAPDH* (glyceraldehyde-3-phosphate dehydrogenase), *HPRT1* (hypoxanthine phosphoribosyl-transferase 1), *RPLP0* (Ribosomal protein lateral stalk subunit P0), and *TBP* (TATA box binding protein) [23]. In their study, *GAPDH* and *HPRT1* exhibited a high standard deviation and low stability, indicating that these genes should not be selected as reference genes. Furthermore, the authors suggested that researchers could select two or three of the remaining four genes—*ABL1, ACTB, RPLP0,* and *TBP* — for data normalization in RT-qPCR assays [23]. In a subsequent study, the authors found that RT-qPCR assays were more sensitive than nested PCR assays in detecting genetic alterations at the time of diagnosis of acute leukemias [24].

## 4. Correlation of the Genetic Polymorphisms, *rs8259* T/A in the CD147 Gene and HLA DRB1*1501, with the Treatment of Cutaneous T-Cell Lymphomas

The research article by Vašků et al. describes the authors’ study of the genetic polymorphisms, *rs8259* T/A in the CD147 gene, and HLA DRB1*1501, and their correlation with therapy in patients with cutaneous T-cell lymphomas (CTCLs) [25]. CD147 is a transmembrane glycoprotein belonging to the immunoglobulin family that exists in several isoforms, one of which is expressed in many types of human cells, including hematopoietic, epithelial, and endothelial cells [26]. This protein has multiple functions, as reviewed in [26]. It induces extracellular matrix metalloproteinases, which are involved in remodeling the extracellular matrix during inflammatory and neoplastic processes, including hematologic malignancies. CD147 is also involved in metabolic pathways and T-cell activation [26], and it can interact with various intracellular, membrane, and extracellular proteins to promote neoplastic processes [27]. In tumor tissues, CD147 expression is positively correlated with the presence of forkhead box transcription factor P3-expressing regulatory T cells, which suppress immunity against cancer [27]. Vašků et al. reported several clinical correlations with the genotypes, which included homozygous polymorphisms that served as a highly specific marker of disease severity, systemic therapy, and radiotherapy in patients with CTCL [25].

## 5. Clustered Regularly Interspaced Short Palindromic Repeats (CRISPR)-Based Gene Editing Could Prevent Hematologic Malignancies in Patients Harboring a Germline Predisposition to Developing Cancer

The fifth article published in this Special Issue focused on clustered regularly interspaced short palindromic repeats (CRISPR)-based gene editing and its current applications in hematology, with emphasis on inherited germline predisposition to hematological malignancies [28]. This paper had a threefold objective: (1) it chronologically describes the history of the discovery of CRISPR/CRISPR-associated (Cas) 9 protein/single-guide RNA-based gene editing; (2) it briefly describes the current state of clinical research on CRISPR-based gene editing in hematologic diseases, along with the genome editing tools used in the described applications, and (3) it briefly summarizes the known inherited genetic predispositions to hematologic malignancies (reviewed in [29]), describes the progress toward gene therapies in inherited hematologic diseases and bone marrow failure syndromes, and presents clinical situations where CRISPR-based gene editing could be applied to potentially prevent the development of hematologic malignancies in individuals harboring a germline genetic predisposition to developing cancer.

Currently, the only curative treatment for individuals with such a germline predisposition is an allogeneic hematopoietic stem cell transplant (HSCT). Applying gene editing based on CRISPR-based tools in such individuals would require an autologous HSCT performed with genetically edited autologous hematopoietic stem and progenitor cells [28]. To avoid the potential toxicities of an HSCT procedure while still enabling a curative treatment, the in vivo modification of hematopoietic stem cells is being developed [28,30]. In this context, as reported last week, researchers in the United States have achieved a significant milestone by rapidly and successfully developing personalized gene therapy using a base editor through a modified CRISPR-Cas9 protein and single-guide RNA and delivering the treatment in vivo to a seven-month-old infant patient born with a rare urea cycle genetic disorder [31]. That entire gene therapy was developed within six months after the newborn was diagnosed with the genetic disease, and it led to a clinical response [31]. Long-term follow-up is required to assess its safety and efficacy [31]. These remarkable scientific and medical advances are based on many decades of dedicated research by scientists and clinicians in the United States and other countries. Notably, these landmark breakthroughs are not restricted to any specific rare disease; they can be applied to numerous other diseases. Therefore, these advances have generated new hope for many patients with potentially treatable genetic diseases, including those with a genetic predisposition to malignancies, the development of which, in such individuals, could be prevented with further research progress.

## Data Availability

The original contributions presented in this study are included in the article. Further inquiries can be directed to the corresponding author(s).

## Abbreviations

The following abbreviations are used in this manuscript:

CLL	Chronic lymphocytic leukemia
BTK	Bruton tyrosine kinase
AML	Acute myeloid leukemia
LSC	Leukemia stem cell
RT-qPCR	Reverse transcriptase quantitative polymerase chain reaction
CTCL	Cutaneous T-cell lymphoma
CRISPR	Clustered regularly interspaced short palindromic repeats
HSCT	Hematopoietic stem cell transplant

## References

[1] Kansal R. (2023). The World Health Organization (WHO) Classification of Tumors with Emphasis on the Classification of Hematolymphoid Neoplasms. Precision Medicine: Where Are We and Where Are We Going?.

[2] Kansal R. (2023). Diagnosis and Molecular Pathology of Lymphoblastic Leukemias and Lymphomas in the Era of Genomics and Precision Medicine: Historical Evolution and Current Concepts—Part 3: Mature Leukemias/Lymphomas. Lymphatics.

[3] American Cancer Society Key Statistics for Chronic Lymphocytic Leukemia (CLL). https://www.cancer.org/cancer/types/chronic-lymphocytic-leukemia/about/key-statistics.html.

[4] Ponader S., Burger J.A. (2014). Bruton’s tyrosine kinase: From X-linked agammaglobulinemia toward targeted therapy for B-cell malignancies. J. Clin. Oncol..

[5] Fresa A., Innocenti I., Tomasso A., Stirparo L., Mosca A., Iadevaia F., Autore F., Ghia P., Laurenti L. (2024). Treatment Sequencing in Chronic Lymphocytic Leukemia in 2024: Where We Are and Where We Are Headed. Cancers.

[6] Alsouqi A., Woyach J.A. (2025). SOHO State of the Art Updates and Next Questions | Covalent Bruton’s Tyrosine Kinase Inhibitors in Chronic Lymphocytic Leukemia. Clin. Lymphoma Myeloma Leuk..

[7] Tam C., Thompson P.A. (2024). BTK inhibitors in CLL: Second-generation drugs and beyond. Blood Adv..

[8] Mato A.R., Woyach J.A., Brown J.R., Ghia P., Patel K., Eyre T.A., Munir T., Lech-Maranda E., Lamanna N., Tam C.S. (2023). Pirtobrutinib after a Covalent BTK Inhibitor in Chronic Lymphocytic Leukemia. N. Engl. J. Med..

[9] Woyach J.A., Furman R.R., Liu T.M., Ozer H.G., Zapatka M., Ruppert A.S., Xue L., Li D.H., Steggerda S.M., Versele M. (2014). Resistance mechanisms for the Bruton’s tyrosine kinase inhibitor ibrutinib. N. Engl. J. Med..

[10] Wiśniewski K., Puła B. (2024). A Review of Resistance Mechanisms to Bruton’s Kinase Inhibitors in Chronic Lymphocytic Leukemia. Int. J. Mol. Sci..

[11] Woyach J.A., Jones D., Jurczak W., Robak T., Illés Á., Kater A.P., Ghia P., Byrd J.C., Seymour J.F., Long S. (2024). Mutational profile in previously treated patients with chronic lymphocytic leukemia progression on acalabrutinib or ibrutinib. Blood.

[12] Chirino A., Montoya S., Safronenka A., Taylor J. (2023). Resisting the Resistance: Navigating BTK Mutations in Chronic Lymphocytic Leukemia (CLL). Genes.

[13] Mullard A. (2021). Targeted protein degraders crowd into the clinic. Nat. Rev. Drug. Discov..

[14] Hayama M., Riches J.C. (2024). Taking the Next Step in Double Refractory Disease: Current and Future Treatment Strategies for Chronic Lymphocytic Leukemia. Onco. Targets Ther..

[15] Montoya S., Bourcier J., Noviski M., Lu H., Thompson M.C., Chirino A., Jahn J., Sondhi A.K., Gajewski S., Tan Y.S.M. (2024). Kinase-impaired BTK mutations are susceptible to clinical-stage BTK and IKZF1/3 degrader NX-2127. Science.

[16] Zhai Y., Shen H., Wei H. (2024). A Comprehensive Metabolism-Related Gene Signature Predicts the Survival of Patients with Acute Myeloid Leukemia. Genes.

[17] Wei H., Kansal R. (2024). Acute Myeloid Leukemia: A Global Perspective of Epidemiology, Prognosis, Treatment and Outcomes. Acute Myeloid Leukemia: Diagnosis, Prognosis, Treatment, and Outcomes.

[18] (2025). National Comprehensive Cancer Network (NCCN) Clinical Practice Guidelines in Oncology (NCCN Guidelines®). Acute Myeloid Leukemia. https://www.nccn.org/professionals/physician_gls/pdf/aml.pdf.

[19] Boscaro E., Urbino I., Catania F.M., Arrigo G., Secreto C., Olivi M., D’Ardia S., Frairia C., Giai V., Freilone R. (2023). Modern Risk Stratification of Acute Myeloid Leukemia in 2023: Integrating Established and Emerging Prognostic Factors. Cancers.

[20] Kansal R. (2022). Fructose Metabolism and Acute Myeloid Leukemia. Explor. Res. Hypothesis Med..

[21] O’Brien C., Jones C.L. (2025). Unraveling lipid metabolism for acute myeloid leukemia therapy. Curr. Opin. Hematol..

[22] Alaggio R., Amador C., Anagnostopoulos I., Attygalle A.D., Araujo I.B.d.O., Berti E., Bhagat G., Borges A.M., Boyer D., Calaminici M. (2022). The 5th edition of the World Health Organization Classification of Haematolymphoid Tumours: Lymphoid Neoplasms. Leukemia.

[23] Pessoa F.M.C.d.P., Viana V.B.d.J., de Oliveira M.B., Nogueira B.M.D., Ribeiro R.M., Oliveira D.d.S., Lopes G.S., Vieira R.P.G., de Moraes Filho M.O., de Moraes M.E.A. (2024). Validation of Endogenous Control Genes by Real-Time Quantitative Reverse Transcriptase Polymerase Chain Reaction for Acute Leukemia Gene Expression Studies. Genes.

[24] Pessoa F.M.C.d.P., de Oliveira M.B., Barreto I.V., Machado A.K.d.C., Oliveira D.S.d., Ribeiro R.M., Medeiros J.C., Maciel A.d.R., Silva F.A.C., Gurgel L.A. (2024). Nested-PCR vs. RT-qPCR: A Sensitivity Comparison in the Detection of Genetic Alterations in Patients with Acute Leukemias. DNA.

[25] Vašků V., Fialová P., Vašků A. (2024). New Genetic Markers of Skin T-Cell Lymphoma Treatment. Genes.

[26] Spinello I., Labbaye C., Saulle E. (2024). Metabolic Function and Therapeutic Potential of CD147 for Hematological Malignancies: An Overview. Int. J. Mol. Sci..

[27] de la Cruz Concepción B., Bartolo-García L.D., Tizapa-Méndez M.D., Martínez-Vélez M., Valerio-Diego J.J., Illades-Aguiar B., Salmerón-Bárcenas E.G., Ortiz-Ortiz J., Torres-Rojas F.I., Mendoza-Catalán M.Á. (2022). EMMPRIN is an emerging protein capable of regulating cancer hallmarks. Eur. Rev. Med. Pharmacol. Sci..

[28] Kansal R. (2024). The CRISPR-Cas System and Clinical Applications of CRISPR-Based Gene Editing in Hematology with a Focus on Inherited Germline Predisposition to Hematologic Malignancies. Genes.

[29] Kansal R., Rezaei N. (2024). Germline predisposition in hematologic malignancies. Comprehensive Hematology and Stem Cell Research.

[30] Breda L., Papp T.E., Triebwasser M.P., Yadegari A., Fedorky M.T., Tanaka N., Abdulmalik O., Pavani G., Wang Y., Grupp S.A. (2023). In vivo hematopoietic stem cell modification by mRNA delivery. Science.

[31] Musunuru K., Grandinette S.A., Wang X., Hudson T.R., Briseno K., Berry A.M., Hacker J.L., Hsu A., Silverstein R.A., Hille L.T. (2025). Patient-Specific In Vivo Gene Editing to Treat a Rare Genetic Disease. N. Engl. J. Med.

